# People that Deliver: Established to Address the Health Supply Chain Workforce Gap

**DOI:** 10.9745/GHSP-D-23-00366

**Published:** 2025-05-09

**Authors:** Dominique Zwinkels, Andrew Brown, Francis Aboagye-Nyame

**Affiliations:** a People that Deliver/UNICEF, Copenhagen, Denmark.; b Management Sciences for Health, Canberra, Australia.; c Management Sciences for Health, Accra, Ghana.

## Abstract

This commentary details the People that Deliver coalition’s work globally in the area of human resources for supply chain management and examines its role in improving the capacity of the health supply chain workforce in low- and middle-income countries.

## Key Messages

- People that Deliver (PtD), a coalition of more than 30 organizations, is united by its goal of developing a competent, supported, and adequately staffed health supply chain workforce deployed across the public and private sectors within the health system in low- and middle-income countries.
- The PtD coalition fills a void in public health by striving to professionalize the supply chain workforce.
- Through its activities, which span advocacy, convening diverse organizations, creating technical tools and frameworks, and developing leadership capacity, the coalition helps to strengthen health supply chains and ensure access to health commodities. Investing in PtD, therefore, means investing in workforce development in low- and middle-income countries.

## INTRODUCTION

In 2009, the Reproductive Health Supplies Coalition published a white paper,^1^ which, for the first time, drew a direct correlation between poor supply chain performance and the lack of a professionalized health supply chain management (SCM) workforce. In particular, this white paper highlighted the insufficient capacities of supply chain managers in low- and middle-income countries (LMICs) and called on governments, donors, and technical partners to work together to implement initiatives to assist countries in developing a professional cadre of supply chain managers and systematically consider human resources (HR) issues beyond training.

Then, in 2011, 79 institutions convened at the World Health Organization headquarters, pledging their support to strengthen the capacity of and professionalize the health supply chain workforce, and People that Deliver (PtD) was established. Since then, PtD has matured into a coalition of more than 30 organizations, united by the common goal of developing a competent, supported, and adequately staffed health supply chain workforce deployed across the public and private sectors within the health system in LMICs.

It is now understood that HR for SCM in the health sector is critical for creating well-managed and mature health supply chains that enable the access, availability, and appropriate use of quality health commodities.^2^ As the global technical leader in HR for health SCM, PtD is working toward a new paradigm in which the health SCM workforce has an elevated status in the health system, is empowered, and has access to relevant incentives and resources, primarily through SCM professionalization. Professionalization leads to positions becoming more attractive thanks in part to their formal status and appropriate career incentives, leading to a market of individuals who seek supply chain qualifications and increased demand from employers to seek such qualifications. In addition, PtD is striving toward a new HR model in which governments and private-sector organizations seek to employ an appropriately qualified workforce of SCM professionals that is provided from within country and regional labor markets.

Through advocacy and by convening a broad array of stakeholders, PtD is the only international coalition that brings together donor organizations, the private sector, and country governments, as well as a multitude of different development partners, learning and development institutions, professional associations, and nongovernmental organizations to advocate on behalf of health supply chain professionals in LMICs. The support PtD provides to country governments in developing the health supply chain workforce—and thereby strengthening health systems—is integral to achieving the Sustainable Development Goals.

In this commentary, we detail PtD’s objectives and achievements globally in the area of HR for SCM, as well as the challenges it faces, and look at PtD’s role in improving the capacity of the health supply chain workforce in LMICs.

## OBJECTIVES

PtD’s mission is to professionalize the public- and private-sector supply chain workforces in the health systems of LMICs, contributing to improved health outcomes. Its work centers on the following 4 strategic enablers.

PtD’s mission is to professionalize the public- and private-sector supply chain workforces in LMIC health systems.

### Convening Diverse Organizations

PtD brings together organizations and individuals with a broad range of skills and experience, facilitating the development of resources that guide countries as they prioritize the development of the SCM workforce (Box).

BOX People that Deliver and Its Coalition Members

Organizations join the People that Deliver (PtD) coalition voluntarily and free of charge. Coalition members benefit from the opportunities to network and collaborate through coalition activities, exchange the latest information, knowledge, and best practices, and contribute to achieving PtD’s objectives. The small secretariat PtD team leverages the expertise of coalition members to produce resources for country governments and development partners to use. These resources—which include tools, frameworks, and guidelines—are designed to improve supply chain performance by providing system-based approaches to enhance and sustain the supply chain management workforce. While the PtD secretariat leverages the expertise of its members to develop these resources, the members are responsible for implementing the resources in the areas in which they operate.

PtD convenes beyond just its coalition. For example, through the Supply Chain Leaders Forum, PtD facilitates collaboration between multiple donor organizations (including Gavi, the Global Fund, the U.S. Agency for International Development, and the World Bank-hosted Global Financing Facility), and LMICs by convening supply chain leaders from more than 25 countries to help facilitate the transition of health SCM from a donor-supported to a country-driven approach. Likewise, PtD convenes countries and regional and development organizations to assist governments in the implementation of a supply chain professionalization agenda.

### Strengthening Leadership Capacity

Today, a lack of trained, skilled, and motivated professionals in the supply chain workforce persists,^3^ owing in part to ineffective leadership, which has long inhibited health supply chains and hindered the supply of health commodities. The second generation of the Strategic Training Executive Program (STEP 2.0), centrally coordinated by PtD, addresses this problem.

Through STEP 2.0, PtD brings together numerous funding organizations, implementing partners, and experienced private-sector expert coaches to help strengthen the capacities of public-sector health supply chain leaders. The purpose is to usher in a new style of leadership, better able to overcome supply chain challenges and lead national SCM transformation. The program centers on “your transformation challenge,” through which the coaches assist participants in addressing a specific supply chain challenge their organization is facing.

### Developing Technical Tools and Frameworks

PtD develops unique resources that help countries to strengthen the health supply chain workforce.

The Building Human Resources for Supply Chain Management Theory of Change^4^ (TOC), published in 2018, is a framework and assessment tool that analyzes the conditions needed to ensure that workers at every level are performing optimally to fulfill all the necessary functions of an effective supply chain system. It posits that addressing 4 pathways—staffing, skills, working conditions, and motivation—is necessary to optimize HR for effective supply chain management. The TOC is embedded in all PtD’s resources and methodologies and was central to PtD’s 2023 business case for investment^5^ in HR for health SCM, guiding the methodology for data collection and analysis.

The SCM Professionalisation Framework^6^ is PtD’s other flagship resource. Published in 2020, it offers a systematic solution to the problem of an inadequate supply of workers and gaps in competencies. Its implementation approach focuses on catalyzing the whole SCM labor market by intentionally changing the system to align career paths, education, and professional growth with competency requirements in the workplace. This approach aims to adjust the structure and elements of government systems to increase the supply and demand of skilled and qualified SCM professionals.

### Engaging in Advocacy

Since PtD’s inception in 2011, advocating SCM workforce investments to country governments, development partners, and donor organizations has been central to its activities. The PtD Global Indaba conference for public and private health supply chain managers and professionals, development partners, and donor organizations has become one of the coalition’s most influential advocacy tools.

The conference, which debuted in 2022 in Zambia and attracted more than 250 participants, aims to help countries plan, finance, develop, support, and retain the national workforces needed for the effective, efficient, and sustainable management of health supply chains. The conference provides PtD with a multidirectional platform that engages all stakeholders in shaping and influencing the SCM workforce development agenda.

The second Global Indaba conference, held in March 2024, gave the PtD the opportunity to appeal to a new audience in Southeast Asia, which included officials from Thailand’s Ministry of Health. The next conference will be held in Kigali, Rwanda, in September 2025.

PtD’s business case for investment in HR for health SCM^5^ remains a significant evidence base from which to advocate increased investments in the supply chain workforce. The findings showed that most investments in the health supply chain workforce in LMICs have been made in staffing (ensuring supply chain positions are filled) and skills development and that the areas of motivation, including performance management and supportive supervision, and working conditions received little funding from donor organizations. The TOC posits that investments are required in each of the 4 pathways (skills, staffing, working conditions, and motivation) for the supply chain workforce to perform optimally and affect commodity availability at service delivery points and, ultimately, health outcomes.

## ACHIEVEMENTS

Although advocacy remains at the core of PtD’s activities, its role has evolved such that it now works with governments in their SCM professionalization efforts. While the coalition has grown to more than 30 organizations, its influence has also increased, evidenced by the prominence of supply chain workforce development on the agenda of many countries, including Mozambique, Nigeria, and Rwanda.

### Shaping Country Agendas

PtD’s TOC has been used in multiple countries as the blueprint to strengthen the health supply chain workforce. In Ethiopia, following the adoption of the TOC, the Ethiopian Pharmaceutical Supply Service earned silver status from the Bill & Melinda Gates Foundation’s Supply Chain Maturity Model, which denotes a process maturity score of between 60% and 79%. In Rwanda, the implementation of the TOC led to the Ministry of Health prioritizing investments in HR for SCM to improve the availability of skilled cadres and institutionalizing SCM professionalization with the adoption of the SCM professionalization framework.

PtD’s TOC has been used in multiple countries as the blueprint to strengthen the health supply chain workforce.

In June 2024, East African Community (EAC) partner states Burundi, Kenya, Rwanda, South Sudan, Tanzania, and Zanzibar committed to developing an EAC regional strategic framework for SCM professionalization within the following 12 months—a commitment facilitated through PtD. The countries have pledged to create an EAC regional policy on professionalizing the health supply chain workforce and a harmonized regional roadmap. Currently, these countries are developing a professionalization task force, which is stage 2 of the 5 stages detailed in the SCM professionalization framework that serves as the countries’ guiding document.

### Improving Supply Chain Leadership in Countries

More than 600 supply chain leaders from over 40 countries have graduated from STEP. In 2024, more countries and participants have enrolled in the program than ever before, with more than 120 participants from at least 11 countries expected to graduate.

Following their graduation in 2021, the participants from the virtual program implemented in Zambia demonstrated significant growth in all domains of leadership competencies and applied their new skills to address a complex challenge in supply chain performance in their organization.^7^ In 2023, PtD developed and began implementing a monitoring and evaluation tool to track the progress of STEP 2.0 participation that has begun to show early indications of the program’s impact. Monitoring activities showed that in 2023, in Rwanda, participants increased their leadership competencies by 8% on average across all competency ratings over the 6-month period of the program (unpublished data). STEP 2.0’s your transformation challenge provides a direct opportunity to measure the achievement of a range of objectives and outcomes, such as increases in product availability, reductions in stock-outs, reductions in emergency orders from health facilities, and improved inventory planning and tracking. In Rwanda, 60% of participants stated that all or almost all of their outcomes had been achieved (unpublished report).

For the regional STEP 2.0 program in Southeast Asia in 2024, a monitoring and evaluation exercise found evidence of supply chain improvements and determined the extent to which STEP 2.0 had influenced the outcome. The results from this research demonstrate that STEP 2.0 enabled participants to gain the knowledge, skills, and tools to address supply chain inefficiencies and improve service delivery in their supply chains. Early results from monitoring activities show that STEP 2.0 improves teamwork and collaboration; increases alignment, engagement, and mobilization behaviors; and catalyzes observable improvements in SCM systems.

## CHALLENGES

Attracting consistent funding is challenging for PtD. The PtD secretariat applies for restricted donor funding every year for project-specific work; typically, only activities lasting 1 year or less are funded. Every year, PtD must reapply for funding, and its budget for the following year remains uncertain. This funding process hinders continuity and limits the impact that can be made in countries. The small budget does not allow PtD to carry out consistent activities, such as monitoring and evaluation, over time.

To maximize its potential, PtD requires substantial and consistent funding so it can plan for the long term. Given the significance of PtD’s role in advocating for and convening so many significant stakeholders in HR for SCM, ensuring the financial sustainability of the coalition should be a priority for the broader global health supply chain community.

PtD makes a substantial contribution to the development of the health SCM workforce. Examples of its impact include the Global Indaba conference and the knowledge exchange that it brings; the progress made by STEP 2.0 and hundreds of public sector supply chain leaders; and the tools and frameworks, such as the professionalization framework and the TOC, that help countries to adequately staff their supply chain workforce. The health SCM workforce and supply chains in LMICs would benefit enormously should the PtD secretariat receive adequate and consistent funding.

## CALL TO ACTION

The confluence of a range of stakeholders through PtD—all with the mutual goal of professionalizing the public- and private-sector supply chain workforce in the health systems of LMICs—has led to significant improvements in country contexts. We urge those with a mandate to strengthen health supply chain systems to use, implement, and share the tools and evidence generated by PtD as they prioritize local interventions. We also encourage readers to engage with PtD by sharing their experiences and lessons learned—this will help us to continue to evolve our collective knowledge.

## Notes

**Peer Reviewed**

**First Published Online:** October 7, 2024.
